# Identification of *Malassezia* species using direct PCR- sequencing on clinical samples from patients with pityriasis versicolor and seborrheic dermatitis

**DOI:** 10.18502/cmm.6.3.3984

**Published:** 2020-09

**Authors:** Mahnaz Gholami, Fatemeh Mokhtari, Rasoul Mohammadi

**Affiliations:** 1 Department of Medical Parasitology and Mycology, School of Medicine, Isfahan University of Medical Sciences, Isfahan, Iran; 2 Skin Diseases and Leishmaniasis Research Center, Department of Dermatology, School of Medicine, Isfahan University of Medical Sciences, Isfahan, Iran; 3 Infectious Diseases and Tropical Medicine Research Center, Isfahan University of Medical Sciences, Isfahan, Iran

**Keywords:** *Malassezia* species, Pityriasis versicolor, Seborrheic dermatitis, 26S rDNA sequencing

## Abstract

**Background and Purpose:**

*Malassezia* yeasts are lipophilic normal flora of the skin in humans and other warm-blooded vertebrates. This genus includes 18 species and is responsible for dermatological disorders, such as pityriasis versicolor, atopic dermatitis, seborrheic dermatitis, folliculitis, and dandruff. The aim of the present study was to identify the etiologic agents of *Malassezia* infections among the patients referring to the Referral Dermatology Clinic of Al-Zahra Hospital, Isfahan, Iran, during 2018-2019.

**Materials and Methods:**

For the purpose of the study, clinical specimens, including skin scrapings and dandruff, were collected and subjected to direct microscopy, culture, and polymerase chain reaction (PCR) sequencing. Direct PCR was performed on the clinical samples to amplify the D1/D2 region of 26S rDNA, using specific primers; subsequently, the amplicons were sent for sequencing.

**Results:**

This study was conducted on 120 patients with suspected pityriasis versicolor and seborrheic dermatitis, who referred to the Referral Dermatology Clinic of Al-Zahra Hospital, Isfahan, Iran, during 2018-2019. Out of this population, 50 (41.7%), 26 (52%), and 24 (48%) cases had *Malassezia* infection, pityriasis versicolor, and seborrheic dermatitis, respectively. *Malassezia globosa* was found to be the most prevalent species (n=29, 58%), followed by *M. restricta* (n=20, 40%), and *M. arunalokei* (n=1, 2%).

**Conclusion:**

The epidemiologic study was indicative of the frequency of some *Malassezia* species, such as *M. globosa* and *M. restricta*, in Isfahan, Iran. It can be concluded that direct PCR on clinical samples could be used as a simple, precise, effective, fast, and affordable method for research and even routine medical mycology laboratory studies.

## Introduction

*Malassezia* species are lipophilic commensal yeasts found as normal skin flora in 70-95% of healthy individuals. This species is also responsible for dermatological conditions, such as pityriasis versicolor (PV), atopic dermatitis, seborrheic dermatitis (SD), folliculitis, and dandruff [ 1 ]. *Malassezia* genus consists of 18 species, among which *M. furfur*, *M. obtuse*, *M. globosa*, *M. slooffiae*, *M. sympodialis*, and *M. restricta* frequently cause human infection. Additionally, *M. pachydermatis*, a zoophilic specie, and *M. dermatis* have been occasionally isolated from human diseases [ 2 ]. These animal-associated species can cause complicated disseminated fungal infections mainly in neonates, immunocompromised infants, and young children candidate for indwelling catheter [ 3 ].

For the first time, Gaitanis et al. [ 4 ] extracted *Malassezia* DNA from skin scrapings using hexadecyltrimethylammonium bromide and identified *Malassezia* species directly from the clinical samples by ITS primers. Since *M. restricta* and *M. obtusa* are fastidious, the isolation of these species from culture media cannot be indicative of real normal flora. Several epidemiological studies on *Malassezia*-related infections have been performed in Iran. For example, Zarei Mahmoudabadi et al. [ 5 ] determined the frequency of the prevalent *Malassezia* species in patients with SD and PV using the nested polymerase chain reaction (PCR) method, in Ahwaz, Iran. They reported *M. obtusa* as the most common species (51.3%), followed by *M. globosa* (35.2%) and *M. restricta*. With this background in mind, the present study was performed to identify the etiologic agents of PV and SD among the patients referring to the Referral Dermatology Clinic of Al-Zahra Hospital, Isfahan, Iran, during 2018-2019.

## Materials and Methods

**Patients**

Patients with hypo- or hyper-pigmented scaly patches, red skin, and dandruff involving the oily regions of the body, who were suspected to have PV or SD were included in the study during June 2018 to November 2019. On the other hand, those with disseminated skin inflammation or skin peeling or the individuals who had taken antifungal agents within the last 2 months were excluded from the study. The study was approved by the Ethics Committee of Isfahan University of Medical Sciences, Isfahan, Iran (IR.MUI.MED.REC.1397.054).

**Sampling**

Skin scrapings and dandruff were collected from the included patients and then subjected to direct microscopic examination with potassium hydroxide 10%, culture on Dixon's agar (HiMedia, India), and direct PCR sequencing. In addition, a concentrated suspension of positive cultures was prepared and kept at -20°C as a source of specimen.

**Direct polymerase chain reaction for the amplification of D1/D2 region of 26S rDNA**

Genomic DNA was extracted from the skin scrapings and dandruff samples using glass bead and phenol/chloroform techniques [ 6 , 7 ]. Briefly, a loopful of the skin scale or dandruff was transferred to a 2-mL Eppendorf tube, containing 300 μL lysis buffer (200 mM Tris/HCl with a pH of 7.5, 25 mM EDTA, 0.5% SDS, and 250 mM NaCl) and 300 μL glass beads. Subsequently, the samples were centrifuged for 1 min at 6,000 rpm for three times and then added with 300 μL phenol/chloroform, followed by vortexing and centrifugation for 5 min at 5,000 rpm. In the next stage, the supernatant was transferred to a new tube, added with the same amount of chloroform, and centrifuged for 5 min at 5,000 rpm. After transferring the superna- tant to a new tube and adding alcohol (2.5 times) and 3 M sodium acetate (1/10 volume), the final mixture was stored at -20°C for 1 h and then centrifuged for 5 min at 10,000. Following the removal of the supernatant, 500 μL alcohol 70% was added to the pellet, which was then centrifuged for 10 min at 10,000 rpm. At this stage, the supernatant was thrown away again, and 50 μL double distilled water was added and kept at -20°C. The PCR amplification was performed at a final volume of 50 µl. Each PCR reaction included 5 µl of 10×PCR buffer, 0.2 mM of each deoxynucleoside triphosphate, 0.5 mM of each forward (5΄- TAACAAGGATTCCCCTAGTA-3΄) and reverse (5΄- ATTACGCCAGCATCCTAAG-3΄) primers [ 8 ], 1.25 U of Taq polymerase, and 2 µl template DNA. The PCR conditions consisted of an initial denaturation step at 95ºC for 5 min, followed by 32 cycles of denaturation at 95ºC for 45 sec, annealing at 55ºC for 45 sec, and extension at 72ºC for 1 min, with a final extension step of 72ºC for 7 min [ 8 ]. The amplified products were visualized by 1.5% (w/v) agarose gel electrophoresis in TBE buffer, stained with ethidium bromide (0.4 µg/ml), and photographed under ultraviolet transilluminator (UVITEC, UK).

**Polymerase chain reaction sequencing**

All PCR products were subjected to sequence analysis. The amplicons were purified using the ethanol purification method; furthermore, cycle sequencing reactions were performed in a forward direction (Bioneer, South Korea). The sequencing products were analyzed with Chromas 2.4 (https://chromas.software.informer.com/2.4/) and then evaluated using the NCBI BLAST searches against fungal sequences existing in DNA databases (https://blast.ncbi.nlm.nih.gov/Blast.cgi).

**Statistical analysis**

The results were analyzed by the Chi-square and Fisher’s exact tests in the SPSS software, version 23 (Armonk, NY: IBM Corp). A p-value less than 0.05 was considered statistically significant.

## Results

A total of 120 patients suspected of PV and SD referred to the Referral Dermatology Clinic of Al- Zahra Hospital from June 2018 to November 2019. Out of 120 subjects, 50 (41.6%), 26 (52%), and 24 (48%) cases had *Malassezia*-related infection, PV, and SD, respectively. The age range of the patients was between 9 and 60 years with the median age of 30.2 years. The age ranges of 21-30 (44%) and 0-10 (2%) years had the highest and lowest frequency, respectively. The male to female ratio of the study participants was 35/15. With regard to the occupational status, the majority of the patients were shopkeepers (22%), students (18%), housekeepers (18%), employees (12%), and construction workers (10%).

**Figure 1:**
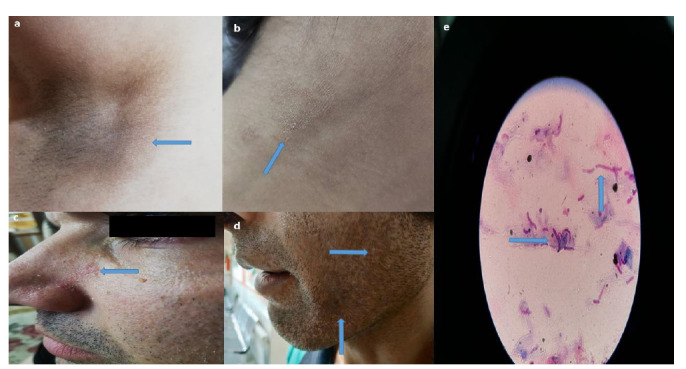
Clinical signs of infection among patients; a) lesions of the armpits, b) lesions on the neck, c) flakes around the sides of the nose, d) flakes of beard, and e) hyphae and globose yeast cells (spaghetti and meatballs) (typical microscopy for *Malassezia* species, Gram stain, original magnification ×100)

**Figure 2:**
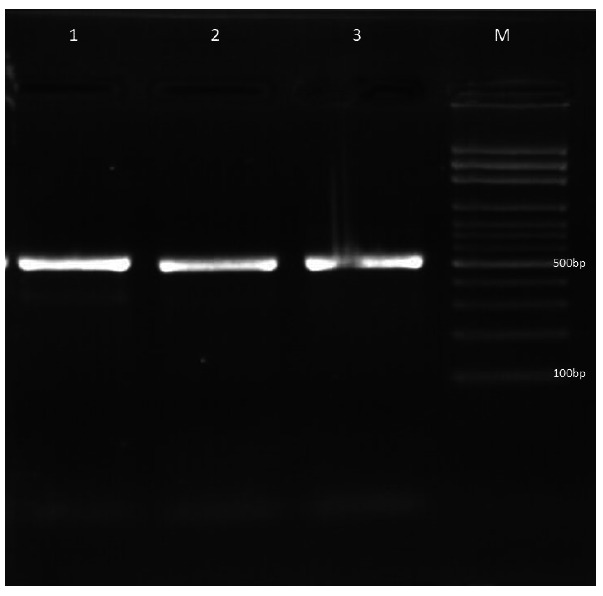
Gel electrophoresis of 26S rDNA-polymerase chain reaction amplicons of *Malassezia* species; lanes 1-3) *M. restricta* and lane M) 100-bp DNA size marker

The clinical specimens were obtained from dandruff (28%), lesions on the neck (24%), lesions on the chest (12%), flakes behind the ears (8%), lesions on the upper back (6%), flakes around the sides of the nose (6%), lesions on the arms (4%), lesions of the armpits (4%), flakes of the eyebrows (4%), groin (2%), and flakes of the beard (2%; Figure 1). The predisposing factors were identified as hyperhidrosis (24%), stress (14%), wearing tight clothes (10%), poor hygiene (8%), solid organ transplantation (2%), and overweight (2%; Table 1). In addition, *Malassezia* *globosa* was found to be the most prevalent species (n=29, 58%), followed by *M. restricta* (n=20, 40%), and *M. arunalokei* (n=1, 2%; Table 1, Figure 2). The results of the Fisher’s exact test showed that the association between the clinical sample type and *Malassezia* species was not statistically significant (P=0.87).

**Table 1:** Demographic and clinical characteristics of patients with different types of Malassezia infection

No.	Gender	Age (years)	Occupation	Primary diagnosis	Predisposing factors	Seasonal distribution	Malassezia spp.	Accession number
1	Male	35	Construction worker	SD	Unknown	Summer	*M. restricta*	MT645538
2	Female	28	Housekeeper	PV	Hyperhidrosis	Summer	*M. globosa*	MT645539
3	Male	26	Shopkeeper	SD	Poor hygiene	Summer	*M. restricta*	MT645540
4	Male	26	Shopkeeper	PV	Poor hygiene	Summer	*M. globosa*	MT645541
5	Female	30	Employee	PV	Stress	Summer	*M. globosa*	MT645542
6	Male	32	Construction worker	PV	Hyperhidrosis	Summer	*M. restricta*	MT645543
7	Male	35	Shopkeeper	SD	Wearing tight clothes	Summer	*M. restricta*	MT645544
8	Male	25	Free-lancer	SD	Stress	Summer	*M. restricta*	MT645545
9	Male	30	Shopkeeper	PV	Hyperhidrosis	Summer	*M. restricta*	MT645546
10	Male	20	Athlete	PV	Wearing tight clothes	Summer	*M. globosa*	MT645547
11	Male	22	Shopkeeper	SD	Unknown	Summer	*M. restricta*	MT645548
12	Male	31	Shopkeeper	SD	Wearing tight clothes	Summer	*M. restricta*	MT645549
13	Male	35	Construction worker	SD	Unknown	Spring	*M. restricta*	MT645550
14	Male	40	Employee	SD	Stress	Summer	*M. globosa*	MT645551
15	Female	38	Housekeeper	PV	Wearing tight clothes	Spring	*M. restricta*	MT645552
16	Male	28	Student	SD	Stress	Spring	*M. globosa*	MT645553
17	Female	30	Housekeeper	SD	Unknown	Fall	*M. restricta*	MT645554
18	Female	29	Housekeeper	SD	Unknown	Summer	*M. globosa*	MT645555
19	Male	17	Student	SD	Unknown	Summer	*M. restricta*	MT645556
20	Male	13	Student	PV	Stress	Summer	*M. restricta*	MT645557
21	Male	54	Shopkeeper	PV	Unknown	Summer	*M. globosa*	MT645558
22	Female	32	Housekeeper	SD	Unknown	Spring	*M. restricta*	MT645559
23	Male	27	Free-lancer	SD	Unknown	Fall	*M. globosa*	MT645560
24	Male	23	Student	SD	Stress	Fall	*M. arunalokei*	MT645561
25	Male	27	Employee	SD	Unknown	Summer	*M. restricta*	MT645562
26	Female	24	Student	SD	Unknown	Summer	*M. globosa*	MT645563
27	Male	29	Shopkeeper	PV	Unknown	Fall	*M. restricta*	MT645564
28	Male	25	Unemployed	SD	Hyperhidrosis	Spring	*M. globosa*	MT645565
29	Male	60	Retired	PV	Solid organ transplantation	Spring	*M. globosa*	MT645566
30	Female	31	Employee	PV	Hyperhidrosis	Spring	*M. globosa*	MT645567
31	Male	35	Shopkeeper	SD	Unknown	Spring	*M. restricta*	MT645568
32	Male	30	Free-lancer	SD	Unknown	Summer	*M. globosa*	MT645569
33	Male	26	Unemployed	SD	Unknown	Summer	*M. globosa*	MT645570
34	Male	34	Construction worker	PV	Poor hygiene	Winter	*M. globosa*	MT645571
35	Male	13	Student	SD	Stress	Fall	*M. restricta*	MT645572
36	Male	27	Driver	SD	Unknown	Fall	*M. restricta*	MT645573
37	Female	33	Housekeeper	PV	Unknown	Summer	*M. globosa*	MT645574
38	Female	30	Employee	PV	Unknown	Spring	*M. globosa*	MT645575
39	Female	32	Housekeeper	PV	Hyperhidrosis	Spring	*M. globosa*	MT645576
40	Male	38	Driver	PV	Unknown	Winter	*M. globosa*	MT645577
41	Male	39	Shopkeeper	PV	Hyperhidrosis	Summer	*M. globosa*	MT645578
42	Female	38	Housekeeper	PV	Overweight	Winter	*M. globosa*	MT645579
43	Male	38	Construction worker	PV	Hyperhidrosis	Fall	*M. globosa*	MT645580
44	Female	23	Student	PV	Hyperhidrosis	Fall	*M. globosa*	MT645581
45	Male	53	Shopkeeper	PV	Wearing tight clothes	Fall	*M. globosa*	MT645582
46	Female	32	Sports Coach	PV	Hyperhidrosis	Fall	*M. globosa*	MT645583
47	Male	9	Student	PV	Poor hygiene	Spring	*M. globosa*	MT645584
48	Female	32	Housekeeper	PV	Hyperhidrosis	Spring	*M. globosa*	MT645585
49	Male	30	Employee	PV	Hyperhidrosis	Spring	*M. globosa*	MT645586
50	Male	15	Student	SD	Unknown	Spring	*M. restricta*	MT645587

PV: Pityriasis versicolor; SD: Seborrheic dermatitis

## Discussion

*Malassezia* yeasts are lipophilic normal microbial flora needing specific lipids, such as oleic acid, for their growth. Colony formation begins quickly after birth and remarkably increments with the increase of neonatal age. Skin colonization by *Malassezia* species is increased from 7% at the first week to 40% at 3-5 weeks of life [ 9 ]. *Malassezia* genus has undergone many taxonomic classifications during the last 2 decades. Up to 1995, three *Malassezia* species, containing *M. obtusa*, *M. sympodialis*, and *M. pachydermatis*, had been identified [ 10 ].

Guého et al. [ 11 ] revealed a taxonomic revision that resulted in four new *Malassezia* species, including *M. obtuse*, *M. globosa*, *M. slooffiae*, and *M. restricta*, based on ultrastructure, physiology, morphology, and also molecular information of rDNA sequencing. Thereafter, other new species were isolated and identified from humans (*M. japonica* [ 12 ], *M. dermatis* [ 13 ], M. yamatoensis) [ 14 ], as well as animals (*M. nana* [ 15 ], *M. equine* [ 16 ], *M. caprae* [ 16 ], and *M. cuniculi* [ 17 ]). Furthermore, four undifferentiated phylotypes were offered in psoriatic and healthy individuals [ 18 , 19 ].

Recently, new *Malassezia* species have been isolated from opossum and parrots based on multilocus sequence analysis [ 20 ], suggesting that *Malassezia* genus may comprise more species than presently known. Similar to the present study, Prohic et al. [ 21 ] and Rodoplu et al.

reported *M. globosa* as the predominant *Malassezia* species isolated from PV lesions in India (52%) and Turkey (65%), respectively. In the same vein, Rasi et al.

reported *M. globosa* as the most prevalent *Malassezia* species in Tehran, Iran, from 2006 to 2007. In the mentioned study, *M. obtusa* was found to be the second most frequent species; however, *Malassezia* furfur was not isolated from Isfahan samples in the current study. Rasi et al. used phenotypic tests, such as Tween assimilation test, splitting of esculin, and catalase reaction, for the identification of the clinical isolates. In the mentioned research, 30.2% of the *Malassezia* culture was negative, which is similar to the result obtained in the present study (32%).

*Malassezia restricta* and *M. globosa* are the species most repeatedly found on the skin of healthy and infected humans. However, other species, such as *M. obtusa* and *M. sympodialis*, have been also associated with different human skin disorders [ 24 , 25 ]. *Malassezia* *globosa* is found more regularly on the arms and chest, whereas *M. restricta* is reported to be more common on the forehead and scalp [ 21 , 26 ]. Furthermore, *M. restricta* has been isolated more frequently in teenagers and young people, while *M. globosa* is the most prevalent species in individuals over 50 years of age [ 27 ]. In many studies, *Malassezia* species are identified based on microscopic characteristics, colony morphology, and complicated and time-consuming tests (e.g., esculin hydrolysis tests, assimilation of Tween 20, 40, and 80, and Cremophor El) [ 28 , 29 ].

Nevertheless, for the first time, Gaitanis et al. [ 4 ] used the nested PCR, PCR-restriction fragment length polymorphism (RFLP), and PCR sequencing for the identification of *Malassezia* species isolated from skin scrapings. They extracted *Malassezia* DNA from 45.5% of samples; however, in the present research, the successful extraction rate from clinical samples was obtained as 100%. The PCR-based method is less costly and more rapid in comparison to the phenotypic methods. Moreover, the traditional methods for *Malassezia* identification are complex and require experienced specialists.

Tarazooie et al. [ 30 ] compared the distribution of *Malassezia* species isolated from PV lesions and those isolated from healthy skins by phenotypic tests. Similar to our study, they reported M. globose as the most prevalent species; however, contrary to our study, they did not isolate any *M. restricta* from the infected patients. The PV is uncommon in children and the elderly. Accordingly, in the present study, there were just one case of PV in a child aged < 10 years and two cases of PV in the subjects aged > 50 years. This result is completely in line with those obtained by Tarazooie et al. [ 30 ].

In another study, Berenji et al. [ 31 ] obtained a prevalence of 58.1% for the superficial fungal infections caused by *Malassezia* species in Mashhad, from 2000 to 2011. In the mentioned study, PV and SD were observed in 19% and 58.4% of the cases, respectively. In the present research, one *M. arunalokei* isolate was isolated from the scalp of a 23-year-old student. He was very stressful because of his lessons and had a long history of dandruff. *Malassezia* arunalokei was described as a new species by Honnavar et al. [ 32 ] in 2016. They observed sequence divergence in the D1/D2 domain, intergenic spacer 1 region of rDNA, ITS region, and the TEF1 gene of new species. Similar to their findings, in the present study,

*M. arunalokei* was isolated from a patient with SD. To the best of our knowledge, the current study is the first report on the isolation of this species from Iran.

## Conclusion

As the findings indicated, the two-step molecular technique adopted in this study could facilitate the identification of all *Malassezia* species without any need for cultivating the clinical isolates. This technique only involved one PCR reaction and a sequencer to identify the clinical isolates at the species level. Accordingly, it can be concluded that this technique can be used as a simple, precise, effective, fast, and affordable method for research and even routine medical mycology laboratory studies.
